# Supplementary material for: Comparison of Orthognathic Surgery Outcomes Between Patients With and Without Underlying High-Risk Conditions: A Multidisciplinary Team-Based Approach and Practical Guidelines
Source: J Clin Med. 2019 Oct 23;8(11):1760. doi: 10.3390/jcm8111760 (PMC6912447; doi:10.3390/jcm8111760)
Supplement: Supplementary file 1 [file jcm-08-01760-s001.zip › Table 1 (Sup. Mater. 1).docx]

**Table 1.** Comprehensive Multidisciplinary Team-Based Orthognathic Surgery Approach.

| **Parameters** | **Characteristics** |
| --- | --- |
| **Multidisciplinary**  **team** | - Anesthesiologists - Plastic surgeons - Orthodontics - Craniofacial nurse specialists - Social workers - Psychologists - Internal medicine specialists |
| **Preoperative care** |  |
| Patient counselling | - Realistic expectations for facial and dental outcomes - Recovery process - Potential complications |
| Surgical-orthodontic  approach | - Screening for diagnosed or presumed underlying conditions of high risk - Accurate diagnosis of skeletal, dental, and facial soft tissue aspects - Surgery-first approach - 3D simulation with definition of bone movements and possible sites of premature bone contact |
| Anesthetic approach | - Assessment of underlying conditions - ASA recommendations - Autologous blood transfusion planning |
| Medical specialists’ approach | - Accurate diagnosis of underlying conditions - Preoperative optimisation - Provide condition-specific guidelines with practical recommendations for perioperative care |
| **Intraoperative care** |  |
| Anesthetic approach | - Videoscope-guided nasal intubation, antibiotics, tranexamic acid, hypotensive anesthesia, regional blocks, local anesthesia, warming control/devices, autologous blood transfusion - Extubation in operative room before transfer to the postanesthesia care unit - Additional measures * |
| Surgical approach | - Single-splint technique - Two-jaw surgery technique - Genioplasty, bone segmentations, and third molar removal - Patient specific 3D-printed surgical splints and repositioning guides - Real-time surgical navigation |
| **Postoperative care** |  |
| Hospital stay | - Postanesthesia care unit to regular wards - Prevention of nausea and vomiting - Dexamethasone 5mg IV q8h for 1 day - IV Cefazolin 1g IV q8h plus Clindamycin 300mg IVF q8h for 2 days - No intermaxillary fixation - No nasogastric tubes - Early mobilization, oral nutrition, and removal of catheters - Intraoral drains (1 day) - Intensive oral hygiene and wound care - Additional measures * |
| Discharge planning | - Ibuprofen 400mg QID - Amoxicillin 500mg PO q8h for 5 days - Patient education for signs and symptoms related to the underlying conditions, oral hygiene, wound care, and nutrition - Scheduled postoperative visits - Additional measures* |
| Early postop  follow-up | - Telephone-based hospital visit within 1st week - Outpatient surgical office follow-up postop day 7 - Outpatient orthodontic office follow-up postop day 14 (x-ray and CBCT for control of the condyle and rigid fixation positions) - Additional measures (postop visits with specialists and complementary exams) * |
| 3D, three-dimensional; CBCT, cone beam computed tomography; postop, postoperative; QID, 4 times a day;  *, specifically implemented for each underlying high-risk condition according to practical guidelines provided by medical specialists (see supplementary Table 2) | |
