# Supplementary material for: Comparison of Orthognathic Surgery Outcomes Between Patients With and Without Underlying High-Risk Conditions: A Multidisciplinary Team-Based Approach and Practical Guidelines
Source: J Clin Med. 2019 Oct 23;8(11):1760. doi: 10.3390/jcm8111760 (PMC6912447; doi:10.3390/jcm8111760)
Supplement: Supplementary file 1 [file jcm-08-01760-s001.zip › Table 2 (Sup. Mater. 2).docx]

**Table 2.** Condition-Specific Practical Guidelines for Perioperative Care.

| **Underlying conditions** | **Preoperative care** | **Intraoperative care** | **Postoperative care** |
| --- | --- | --- | --- |
| - **Hashimoto's thyroiditis** | - Check thyroid hormone level, and TSH level - Prevent hypothyroidism - Check Potassium levels | - Intraoperative checking of potassium levels to prevent type 1 renal tubular acidosis (caused by loss of K) | - The fluid loss and replacements intraoperatively paired with the loss of K in thyroiditis can lead to K imbalance. Watch out for signs of hypokalemic periodic paralysis which can manifest as transient episodes of flaccid muscle weakness |
| - **Anemia** | - Check Hb level - Correct Hb level with transfusion with pRBC prior to operation | - Transfusion with pRBC 2u prn if bleeding > 1000ml or hemodynamic unstable | - Post-transfusion complete blood count to monitor the need for either iron supplement or another transfusion |
| - **Thrombocytopenia** | - Check platelet count - Transfusion with leukocyte-poor platelet 12u prior to operation | - Transfusion with leukocyte-poor platelet 12u prn if prolonged operation or easily bleeding | - Post-transfusion complete blood count to determine the platelet level postoperatively and the possible need for another transfusion |
| - **Dermographism Urticaria** | - Antihistamines preoperatively - Prophylactic Prednisone 12hrs and 1 hour before surgery - Midazolam to attenuate stress-induced exacerbation - Careful handling of patient to prevent offending stimuli | - Avoid histamine releasing drugs - Strict blood pressure monitoring - WOF: bronchospasm and hypotension | - Continue prednisone up to 3 days postoperatively |
| - **Diabetes mellitus (DM)** | - Check AC/PC sugar, creatinine - Check HbA1C if not measured in previous 3 months - Do ECG - Keep routine antidiabetic drugs until the morning of operation | - Check finger sugar regularly during NPO - Evaluate supplemental insulin use - Avoid marked hyperglycemia - Avoid hypoglycemia | - Evaluate the restarting of routine antidiabetic drugs if eating well - Avoid marked hyperglycemia - Avoid hypoglycemia |
| - **Hyperthyroidism** | - Check TSH, free T4, T3 levels - Evaluate clinical severity and thionamides/iodine treatment - Give beta-blockers in the absence of contraindications | - Watch out thyroid storm | - Watch out thyroid storm in the first 18 hours after operation - Keep thionamides and beta-blockers treatment |
| - **Hypothyroidism** | - Check TSH, free T4 levels - Check cortisol level if adrenal insufficiency is suspected - Evaluate clinical severity and thyroxine supplement | - Watch out intraoperative hypotension and extreme sensitivity to opioids, sedatives, and anesthesia in severe cases | - Keep thyroxine supplement as preoperative care |
| - **Hypopituitarism** | - Determine preoperative hormonal status - Test functional gland reserve using stimulation and suppression tests - Check the chronic therapy that can influence the choice of perioperative drugs - Prepare for difficult intubation - Stress dose hydrocortisone replacement prior to surgery | - Watch out for hypersensitivity reactions to sedatives and general anesthesia - Strict monitoring of cardiovascular function - Patients are prone to hypoglycemia and fluid and electrolyte imbalances (strict intraoperative monitoring and hourly diuresis and fluid balance measurement) | - Postoperative determination of electrolyte and glucose levels to ensure normal levels after fluid shifts after surgery - Reinstallment of normal doses of hormonal therapy which could have been tapered during surgery |
| - **Transgender** | - Preanesthetic evaluation - Risk assessment of hormone replacement therapies, including laboratory testing: - Many laboratory values are calculated utilizing the case’s biologic sex for pharmacological computations - Some tests should be ordered based on biological sex. eg, check PSA for MtF, check hCG for FtM - Psychiatric consultation / Psychological assessment | - Prevent deep vein thrombosis and pulmonary embolism (MtF) - Careful airway management during intubation (MtF) | - Psychiatric follow-up for the evaluation of psycho-social issues and emotional impacts, such as anxiety and depression. - Team approach: support the case’s mental health by pharmacological and nonpharmacological interventions |
| - **VSD, RVOT s/p** | - ECG, echocardiography for cardiac function | - Intra-operative hemodynamic stable | - Monitor vital signs |
| - **PDA, ASD s/p** | - ECG, echocardiography for cardiac function | - Intra-operative hemodynamic stable | - Monitor vital signs |
| - **Osteogenesis imperfecta (OI)** | - CT, X-ray image study - Aware that bones fracture easily - * Prepare for difficult intubation (due to bone deformities) | - Difficult intubation, cervical instability - One jaw (BSSO) surgery is preferred | - Osteotomy healing |
| - **Glycogen storage disease** | - Glycemic level determination and correction of glycemic imbalance - Thorough assessment of cardiac and pulmonary functions - Risk for post-operative sudden hemodynamic collapse | - Close monitoring of glucose levels - Use of glucose containing fluids - Watch out for metabolic acidosis | - Postoperative determination of acid-base balance and glucose levels. Correct imbalances as needed |
| - **Wilson’s disease** | - Thorough neurological and psychiatric evaluation (Pre-existing neuropsychoological problems can be aggravated by hypnotic and sedative drugs) - Preoperative complete blood count (copper accumulation can lead to erythrocyte hemolysis) - Liver function tests including ammonia and coagulation factors | - Be wary of the choice and dose of anesthetic drugs (patients have decreased hepatic metabolism and renal excretion, and altered neuromuscular transmission) - Prepare for delayed emergence after general anesthesia (due to dysfunction in hepatic metabolism) - Careful use of non-depolarizing neuromuscular blocking agents (patients have increased sensitivity) | - Thorough neuromuscular physical examination after full emergence from anesthesia - Postoperative complete blood count (rule out hemolytic anemia, correct as needed) |
| - **Marfan syndrome** | - Careful evaluation of the cardiovascular system (especially aortic aneurysm and mitral valve prolapse) - Pulmonary evaluation (prone to spontaneous pneumothorax and pulmonary emphysema) - Endocarditis prophylaxis for those with prosthetic cardiac valve | - Adequate ventilator pressures to prevent rupture of emphysematous bullae - Hemodynamic monitoring (prone to dysrhythmias) - Gentle handling of patient (joints prone to subluxation and dislocations) | - Postoperative cardiopulmonary evaluation |
| - **Biliary atresia** | - Check liver function, coagulation, platelet count - Prophylactic splenectomy or embolization of spleen (for patients with splenomegaly) - Check respiratory reserve for cirrhotic patient due to ascites | - Maintain hemodynamic stable - Bleeding control (prone to coagulopathy) | - Watch for thrombocytopenia - Observe drug metabolism or durg-drug interaction |
| - **Spinal muscular atrophy (SMA)** | - Meticulous preoperative airway evaluation to determine the best intubation technique individualized for the patient depending on the problem present (limited mobility of cervical spine, joint contractures, etc.) - Prepare for difficult intubation | - Careful use of nondepolarizing neuromuscular blockers (SMA patients have prolonged effect of this drug) - Avoid succinylcholine - Careful titration of opioids - Careful respiratory monitoring | - Postoperative pulmonary physiotherapy to prevent respiratory complications |
| - **Autosomal dominant polycystic kidney disease (ADPKD)** | - Good blood pressure control - Preoperative determination and correction of electrolyte imbalance - Preoperative complete blood count to rule out thrombocytopenia, and subsequent transfusion of platelets if indicated | - Meticulous fluid balance monitoring - Avoid using drugs that cause cardiovascular depression - Strict blood pressure monitoring | - Postoperative electrolyte determination and complete blood count |
| - **Sturge Weber syndrome** | - Informed of hard tissue and soft tissue problems, staged surgery - Contrast CT angiography for possible intracranial lesions - Neurologist or ophthalmologist consultation is necessary if epilepsy or glaucoma. | - Difficult intubation due to peri-oral lump - Minimize the potential risk of seizure - Careful choice of appropriate anesthetic agents, anticonvulsants, and pharmacologic manipulation related to intraocular and intracranial pressure | - Facial hyperpigmentation remains - Maintain the medication for glaucoma and seizure |
| - **HIV** | - Check hemogram, bleeding tendency, renal function, liver function - Get the data about absolute CD4 count, HIV viral load and lipid profile in recent 3-6 months - Review the medication of HAART - Evaluate the risk of cardiovascular events if the patient with hyperlipidemia and/or smoking | - Avoid contact of patients' blood with the skin and mucous membranes of health care workers - The operators and assistance should be equipped with protective eyewear, masks, water-impermeable gowns, sleeves and boots. Wearing two pairs of latex gloves is necessary. - Maintain hemodynamic stable | - Wound infection, especially in patient with CD4 count< 200 cell/cc - Watch for any other new infection signs or symptoms, especially in patients with CD4 count < 200 |
| - **HBV** | - Check liver function, Coagulation profile, HBV viral load (HBV DNA) - The Child-Pugh score - Liver ultrasound(elastography) - Cardiopulmonary and renal function for cirrhotic patients with hepatorenal and hepatopulmonary syndrome - Preoperative nutrition support - Optional vitamin K support - Correction of coagulopathy prior to operation | - Maintain hemodynamic stable - Minimize blood loss - Optimal preemptive antibiotics for cirrhotic patients - Transfusion with whole blood, FFP, pRBC or platelet if necessary | - Check liver function, Coagulation profile, HBV viral load (HBV DNA) - Monitor potential stress-related HBV hepatitis flare-up - Adequate nutrient supplement for moderate-severe cirrhotic patients for wound recovery - Prevent over-dose opioid agent |
| - **Depression** | - Psychiatric consultation: A stable psychiatric status of the patient is important before surgery - Preoperative electrocardiography should be evaluated considering the side effects of many psychotropic agents (eg, prolonged QTc intervals) - Adjust psychotropic medication regimen to reduce through psychiatric drug direct effect and its interaction with anesthesia, and manage withdrawal symptoms of psychotropic drugs. Generally, SSRIs treatment may be continued | - Electrocardiograph monitoring - Maintain hemodynamic stable - Prevent serotonin syndrome | - Psychiatric consultation for aesthetic change, risk of psychiatric disorder relapse due to stressors during the postoperative period or sudden discontinuation of psychiatric medication - Adjust psychotropic medication regimen - Prevent serotonin syndrome - If the patient is taking SSRIs, risk of intestinal bleeding should have been taken into account at concomitant prescription of NSAIDS |
| - **Systemic lupus erythematosus (SLE)** | - Check disease activity: C3/C4, dsDNA - Cardiac function: blood pressure, peripheral pulses, Complete ECG, echocardiogram [especially for heart failure, pulmonary hypertension] - Pulmonary function: chest radiograph at baseline [a crude assessment for interstitial lung disease and serositis], Pulmonary function test, or possible HRCT - Renal function/electrolyte/Urine protein - CBC-DC: cytopenia   - Hemolytic anemia: Correct anemia before emergency surgery by Leukocyte-poor RBC  - Thrombocytopenia: Correct thrombocytopenia before emergency surgery by Leukocyte-poor platelet (at least 80× 10^3^ /uL in a major operation), except thrombotic thrombocytopenic purpura (TTP) or microangiopathic hemolytic anemia (MAHA)  *Surgery will need to be postponed in case of TTP or MAHA unless the procedure is an important option to remove the cause of TTP or MAHA, such as severe infection or disseminated malignancy. | - Hemodynamic stability motoring | - Wound healing - Mental support - Renal function - Electrolyte - CBC-DC - Steroids resume - NSAIDs resume - Immunosuppressants resume |
| - **SLE with positive autoantibodies for anti-phospholipid syndrome (APS)** | - Check autoantibodies for APS:   Anti-B2-glycoprotein 1 Ab, Anti-phospholipid Antibody IgG, Anti-phospholipid Antibody IgM, Anti-cardiolipin-IgG, Anti-cardiolipin-IgM, Lupus anticoagulant lupus anticoagulant   - Confirm any history of vascular thrombosis event - Prophylactic low molecular weight heparin - Confirm any medication of antiplatelet and anticoagulation - Aspirin: Be stopped for 5 to 7 days prior to surgery and should be restarted 3 to 4 days postoperatively. - Warfarin: Should be held off at least 5 days prior to surgery and replaced by low molecular weight heparin, which should be held off in the morning of surgical procedure. (Optimal perioperative INR < 1.5) | - Hemodynamic stability motoring | - Early mobilization after operation - Keep low molecular weight heparin until the sonographic absence of DVT is proven. - Routinely screening for deep vein thrombosis (DVT) between day 3 and 5 after an operation of SLE patient. - Aspirin: Should be restarted 3 to 4 days postoperatively. - Warfarin: Should be re-commenced as soon as patients are hemodynamically stable with minimal bleeding risk. |
| - **Systemic sclerosis** | - Cardiac function: blood pressure, peripheral pulses, Complete ECG, echocardiogram [especially for right heart failure, pulmonary hypertension] - Pulmonary function: chest radiograph at baseline [a crude assessment for interstitial lung disease and serositis], Pulmonary function test, 6 minutes walking test, DLCO, or possible HRCT - Esophagogastroduodenoscopy to exclude gastroesophageal reflux disease (GERD) and esophageal dysmotility (dysphagia), and preventive antacid or H2-receptor antagonists or Proton-pump inhibitor - Renal function/electrolyte/Urine protein | - Limited mouth opening, difficult intubation - Aspiration preventing - Not exacerbated lung problem - Hemodynamic stability motoring - Avoid the aggravation of Raynaud’s syndrome | - Wound healing - Mental support - Renal crisis monitoring - GI bleeding and intestinal pseudo-obstruction monitoring - Renal function - Electrolyte - Steroids resume - NSAIDs resume - Immunosuppressants resume |
| - **Behçet's disease** | - Check oral condition - Ophthalmological examination for uveitis including dilatation, fundoscopy and slit lamp examination to assess the extent of uveal tissue involvement. - Consider MRA or angiography to evaluate for aneurysms, especially involving the pulmonary and cerebral vasculature. - Avoid skin and mucosa puncture for nerve blocks and other regional procedure, and multiple pricks for intravenous access. [May predispose inflammation and exhibit pathergy with the evolution of papules over 24 - 48 hours] - Review any event of vasculitis - Thrombosis monitoring and preventing | - Intra-oral condition - Avoid supraglottic airway device, and repeated attempts at laryngoscopy and intubation [may lead to post-operation ulcer and nodule formation] - The usage of tourniquet should be guarded and be done only after ruling out DVT by Doppler studies. - Regional anesthesia is contraindicated in patients with CNS Behçet’s disease. | - Wound healing - Mental support - Renal function - Vasculitis monitoring - Routinely screening and prophylaxis for deep vein thrombosis, pulmonary embolism and migration of thrombus - Steroids resume - NSAIDs resume - Immunosuppressants resume |
| - **Steroid in rheumatology disease** | - Glucocorticoids:   - Shift to hydrocortisone 100 mg intravenously on call to the operation theatre   - *Notice that non-corrected adrenal function suppression before an operation during the first 48 hours may develop circulatory shock and renal shutdown, if chronic glucocorticoid administration (e.g., prednisolone 5 mg or equivalent and above for more than 2 weeks). |  | - Glucocorticoids:   - After the operation, hydrocortisone 100 mg intravenously every 8 h on the first day, followed by every 12 hourly and daily on the second and third day. - If stable, oral glucocorticoids of the usual dose can be re-commenced. |
| - **NSAIDs in rheumatology disease** | - NSAIDs:   - Encourage to withhold NSAIDs preoperatively for a period equivalent to five half-lives of the drugs in order to restore normal platelet function. - COX-2 NSAIDs do not affect platelet function and are safe to be given peri-operatively. |  | - NSAIDs: can be re-started 2-3 days postoperatively. |
| - **Immunosuppressants in rheumatology disease** | - Immunosuppressants: - Cease any bone marrow immunosuppressant |  | - Immunosuppressants: - If no active infection, resume the previous drugs step-by-step |
| TSH: thyroid stimulating hormone, K: potassium, Hb: hemoglobin, pRBC: packed RBCs, 2u: 2 units, prn: pro re nata, AC/PC: ante cibum/post cibum, HbA1C: glycosalated hemoglobin, EKG: electrocardiogram, NPO: nothing per os, PSA: prostatic specific antigen, MtF: male to female, hCG: pregnancy strip test, FtM: female to male, CT: computed tomography, BSSO: bilateral sagittal splitting osteotomy, HIV: human immunodeficiency virus, HBV: hepatitis B virus, HAART: highly active antiretroviral therapy, FFP: fresh frozen plasma, SSRI: selective serotonin reuptake inhibitors, HRCT: high resolution computed tomography, NSAID: non-steroidal anti-inflammatory drug | | | |
