# Supplementary material for: Comparison of Orthognathic Surgery Outcomes Between Patients With and Without Underlying High-Risk Conditions: A Multidisciplinary Team-Based Approach and Practical Guidelines
Source: J Clin Med. 2019 Oct 23;8(11):1760. doi: 10.3390/jcm8111760 (PMC6912447; doi:10.3390/jcm8111760)
Supplement: Supplementary file 1 [file jcm-08-01760-s001.zip › Table 3 (Sup. Mater. 3).docx]

**Table 3.** Characteristics of Patients with Underlying High-Risk Conditions.

**No. Sex Age Blood Operative Regular ward Complications Underlying diseases**

**Loss (ml) Time (min) admission (day)**

**Genetic disorders**

1 M 19 400 300 5 no Osteogen imperfecta

2 F 21 1740 315 3 no Osteogen imperfecta

3 F 17 400 263 3 no Glycogen storage disease

4 M 21 450 329 4 no Wilson's disease

5 F 24 880 320 4 no Marfan syndrome

6 M 26 370 238 2 no Spinal muscular atrophy

7 F 18 2050 393 5 no Biliary atresia with liver cirrhosis

8 M 23 1000 371 2 no Autosomal dominant polycystic

kidney disease

**Autoimmune diseases**

9 F 31 700 277 4 no Systemic lupus erythematous

10 F 20 2010 449 5 no Hashimoto thyroiditis

11 F 26 500 543 6 no Systemic sclerosis

12 M 26 1440 392 4 no Bechet's disease

13 M 18 1010 261 4 no Anemia

14 M 29 980 343 4 no Thrombocytopenia

15 F 27 410 360 4 no Dermographism urticaria

16 F 29 1180 476 4 no Type I diabetes mellitus

**Endocrine diseases**

17 F 19 590 194 3 no Hypothyroidism

18 F 24 900 344 3 no Hypothyroidism

19 M 27 1710 388 4 no Hypopituitarism

20 F 19 300 160 3 no Hyperthyroidism

21 M 24 1440 512 5 no Transexual MtF

**Neuro-cutaneous disorders**

22 F 16 2190 492 4 no Sturge Weber syndrome

23 F 31 2000 300 5 no Sturge Weber syndrome

24 F 22 1050 370 4 no Sturge Weber syndrome

**Infection**

25 F 37 610 310 4 no Chronic hepatitis B

26 M 39 1150 497 4 no HIV

**Heart**

27 F 28 1100 494 4 no VSD, RVOT obstruction, PV stenosis

28 F 20 630 377 4 no PDA ASD s/p

**Psychiatric disorder**

29 F 41 350 195 5 no Depression

30 F 29 500 229 3 no Depression

MtF = male to female, HIV = human immunodeficiency virus, VSD = ventricular septal defect, RVOT = right ventricular outflow tract, PV = pulmonary valve, PDA = patent ductus arteriosus, ASD = atrial septal defect
